# Improved crystallographic models through iterated local density-guided model deformation and reciprocal-space refinement

**DOI:** 10.1107/S0907444912015636

**Published:** 2012-06-19

**Authors:** Thomas C. Terwilliger, Randy J. Read, Paul D. Adams, Axel T. Brunger, Pavel V. Afonine, Ralf W. Grosse-Kunstleve, Li-Wei Hung

**Affiliations:** a Bioscience Division and Los Alamos Institutes, Los Alamos National Laboratory, Los Alamos, NM 87545, USA; b Department of Haematology, Cambridge Institute for Medical Research, University of Cambridge, Cambridge CB2 0XY, England; c Lawrence Berkeley National Laboratory, One Cyclotron Road, Building 64R0121, Berkeley, CA 94720, USA; d Departments of Molecular and Cellular Physiology, Neurology and Neurological Science, Structural Biology, Photon Science, and Howard Hughes Medical Institute, Stanford University, 318 Campus Drive West, Stanford, CA 94305-5432, USA; e Physics Division, Los Alamos National Laboratory, Los Alamos, NM 87545, USA

**Keywords:** molecular replacement, automation, macromolecular crystallography, structure similarity, modeling, *Phenix*, morphing

## Abstract

An approach is presented for addressing the challenge of model rebuilding after molecular replacement in cases where the placed template is very different from the structure to be determined. The approach takes advantage of the observation that a template and target structure may have local structures that can be superimposed much more closely than can their complete structures. A density-guided procedure for deformation of a properly placed template is introduced. A shift in the coordinates of each residue in the structure is calculated based on optimizing the match of model density within a 6 Å radius of the center of that residue with a prime-and-switch electron-density map. The shifts are smoothed and applied to the atoms in each residue, leading to local deformation of the template that improves the match of map and model. The model is then refined to improve the geometry and the fit of model to the structure-factor data. A new map is then calculated and the process is repeated until convergence. The procedure can extend the routine applicability of automated molecular replacement, model building and refinement to search models with over 2 Å r.m.s.d. representing 65–100% of the structure.

## Abstract 2

A density-based procedure is described for improving a homology model that is locally accurate but differs globally. The model is deformed to match the map and refined, yielding an improved starting point for density modification and further model-building.

## 1. Introduction

One of the most important methods for determining macromolecular structures is molecular replacement (Rossmann, 1972 ▶). In this procedure, a known structure is used as a template for the target structure to be determined. An approximate position of the template is found, typically using a search procedure that optimizes the agreement between the observed structure factors and those calculated from the placed template (see, for example, Navaza, 1987 ▶; Vagin & Teplyakov, 1997 ▶; Read, 2001 ▶; McCoy *et al.*, 2007 ▶; Keegan *et al.*, 2011 ▶). The placed template is then used to generate a starting electron-density map that can be a basis for model improvement or rebuilding.

A crucial requirement of the molecular-replacement method is that the template be quite similar to the target structure. Usually, these two structures must agree within about 1.5–2 Å root-mean-square distance (r.m.s.d.) for C^α^ atoms over much of the molecules to be useful in molecular replacement (Chen *et al.*, 2000 ▶). This means that the sequences of the template and target usually need to be about 25–30% identical or greater (Chothia & Lesk, 1986 ▶). Despite this limitation, over 70% of new protein structures are already determined by molecular replacement (Evans & McCoy, 2008 ▶). As the number and diversity of structures in the Protein Data Bank (PDB; Berman *et al.*, 2000 ▶) increases, the applicability of molecular replacement will continue to broaden.

The utility of molecular replacement would be extended even further if the requirement for similarity between the template and target structures could be relaxed. Recently, several methods have been introduced that address this requirement. The use of algorithms from the structure-modeling field has yielded improved homology models based on distant templates, improved models obtained from other techniques such as NMR and even *ab initio* models that are suitable for molecular replacement (Qian *et al.*, 2007 ▶; Ramelot *et al.*, 2009 ▶; DiMaio *et al.*, 2011 ▶; Mao *et al.*, 2011 ▶). Additionally, algorithms from the structure-modeling field have been combined with crystallographic tools to rebuild and improve templates that have been placed in position in the crystallo­graphic cell using weak structural information available from initial electron-density maps calculated using these placed templates (DiMaio *et al.*, 2011 ▶). Methods for the iterative improvement of models and electron-density maps have further increased the convergence of molecular replacement, particularly when data are available at resolutions finer than about 2 Å (Perrakis *et al.*, 1999 ▶; Langer *et al.*, 2008 ▶; Cohen *et al.*, 2008 ▶). Finally, techniques that incorporate local structural information from the template as restraints have increased the amount of information available in refinement, facilitating improved refinement at low resolution and refinement starting with models that are more distant from the target structure than was previously feasible. These methods include LSSR in *Buster* (Smart *et al.*, 2008 ▶) and external structure restraints in *REFMAC* (Murshudov *et al.*, 2011 ▶), each of which uses distance restraints between nearby atoms derived from the reference model to inform the refinement. DEN restraints in *CNS* (Schröder *et al.*, 2007 ▶, 2010 ▶) use ‘deformable’ networks of distance restraints, permitting slow deformations of the restraints as the refinement proceeds and adjusting the degree of deformation by cross-validation with *R*_free_ using multiple trials for each parameter combination, ensuring the most optimal refined structure. Other methods include the use of restraints in torsion-angle space derived from the reference model (Headd *et al.*, 2012 ▶) and the use of normal-mode refinement (Kidera & Go, 1992 ▶; Delarue, 2008 ▶).

In this work, we describe a method for iterated local density-guided model deformation and refinement, a process that we will refer to here with the informal term ‘morphing’. Morphing can be applied to search models that have been placed in the crystallographic cell by molecular replacement but that are not close enough to the target structure for automated model building to be effective. Our approach for morphing builds on methods for finding fragments of structure in electron-density maps (Kleywegt & Jones, 1997 ▶; Cowtan, 1998 ▶; Terwilliger, 2001 ▶), but extends these methods by allowing a different translation for each residue in a template, smoothing these translations to yield a continuously deformed model with an improved match to the electron-density map. Further, the morphing procedure includes refinement to improve model geometry and the fit to crystallographic data. We show that morphing can be useful in improving an initial molecular-replacement model after it has been placed in the crystallographic cell.

## 2. Methods

### 2.1. Why morphing of a model might be useful

The reason morphing of a model might be useful is that the local coordinate differences between two homologous structures are often considerably smaller than the global coordinate differences (Holm & Park, 2000 ▶; Schneider, 2002 ▶; Ye & Godzik, 2003 ▶; Roach *et al.*, 2005 ▶). Homologous proteins with sequence identities in the range 20–30% typically have conserved core structures (Chothia & Lesk, 1986 ▶). Despite this overall similarity, the coordinates of segments of secondary-structural elements often still cannot be precisely superimposed owing to variations in the relative positions of these segments. For example, residues in a β-sheet and those in an adjacent α-helix might have similar relationships in two homologous structures, but the precise position or orientation of the sheet relative to the helix could differ. In such a case the β-sheets could be superimposed very closely, or the α-helices could be superimposed closely, but not both simultaneously.

If two structures are very similar at a local level but have differences on a larger scale, then a small number of parameters can be used to deform one structure to match the other much more closely (Ye & Godzik, 2003 ▶). This is most obvious in a case where two structures differ simply by a hinge motion, but the approach is applicable to a variety of types of deformations relating two structures. Importantly, if only a few parameters need to be determined then very weak information averaged over large regions can be used to identify the values of these parameters.

### 2.2. Morphing a model to match an electron-density map

Our procedure for morphing of a model requires a starting model and a starting electron-density map. The procedure consists of three steps. Firstly, for each residue in the model a translation to be applied to atoms in the vicinity of this residue is identified that maximizes the overlap between these atoms and the electron-density map. Next, these translations are smoothed within segments of structure. Finally, the smoothed translation for each residue is applied to each atom in that residue. This can be followed by refinement to improve the geometry and the entire process can be iterated until convergence.

### 2.3. Optimizing a translation to match a group of atoms to a map

An FFT-based procedure (Cowtan, 1998 ▶; Terwilliger, 2001 ▶) is used to identify a translation that best matches the atoms near the C^α^ atom of a given residue to a target electron-density map, with one FFT calculated for each residue. A model-based (*F*_calc_) map is first calculated from all the atoms in the structure within a radius *r*_morph_ of the C^α^ atom of a given residue. For nucleic acids, a similar procedure could be used, centering at the C_1_′ carbon. Here, *r*_morph_ is typically 6 Å for electron-density maps calculated at a resolution of about 3 Å.

To identify appropriate values of *r*_morph_, radii ranging from 3 to 12 Å were tested using the structure hp3342 (described below; PDB entry 3tx8, Brunger *et al.*, 2012 ▶), with only a small effect on the resulting model quality. The map correlation between a 2*mF*_o_ − *DF*_c_ map (Read, 1986 ▶) calculated after morphing to a 2*mF*_o_ − *DF*_c_ map based on the final refined model ranged from 0.58 to 0.65, with the maximum at a radius of 6 Å. Additionally, we tested morphing starting with a large radius (12 Å), decreasing it each cycle to radii from 3 to 9 Å; again, the resulting model quality varied only slightly (map correlation ranging from 0.62 to 0.63). The reason for the relative insensitivity of the results to the radius used is likely to be that the coordinate shifts are smoothed (below) over a much larger region encompassing 11 residues.

The model-based map is set to zero outside *r*_morph_ and a density offset is added to make the mean of the map inside *r*_morph_ equal to zero. The convolution of this map with the target electron-density map is then calculated as described previously (Terwilliger, 2001 ▶). The value at coordinates **x** of the resulting convolution is the overlap integral of the model-based map, offset by **x**, with the electron-density map. The overlap integral of the target electron-density map and the model map is calculated for all possible offsets (the FFT is calculated over the entire unit cell using grids of about 1/4 to 1/3 of the high-resolution limit of the target map). Although this convolution is calculated everywhere in the crystallo­graphic cell, only small offsets |**x**| are plausible. Consequently, the convolution map is examined only in the region within a radius *r*_max_ of the origin (where typically *r*_max_ = 2 Å). The centroid of the highest peak in this region (Δ**x**_cent_), along with the co­ordinates of the grid point with the highest value (Δ**x**_max_), are noted. The initial estimate of the rigid-body offset to apply to this residue is then the centroid (Δ**x**_cent_) of the highest peak in the convolution map that is within *r*_max_ of the origin. A local correlation (cc_local_) between the electron-density map and the model-based map (offset by the grid point with the highest value, Δ**x**_max_) is calculated as well.

At this stage, any possible overlaps between an offset residue and another residue in the structure are ignored, as are any problems owing to the density for one residue giving a high correlation when an adjacent residue is moved there. Although these complications could affect the morphing process, the smoothing stage that follows can potentially remove many problematic cases.

### 2.4. Smoothing residue shifts within contiguous segments

In this procedure, it is assumed that the shifts to be applied to the structure vary gradually along a chain. The vector shifts Δ**x**_max_ for all the residues in a chain are therefore smoothed, typically in a window of 11 residues. A linear regression for the values of Δ**x**_max_, with residue number as the independent variable, is calculated. The linear regression is used instead of a weighted mean because some points may not be included (see below). The value of the smoothed shift for residue *j* is then the value of Δ**x**_max_ estimated from the regression at that residue number. Shifts for residues for which the map correlation (cc_local_) with atoms within a sphere of radius *r*_morph_ is less than a threshold of cc_min_ (typically cc_min_ = 0.05) are not included in the smoothing. An alternative method to smooth the shifts might be to translate a window of residues as a rigid body and then apply the shift only to the residue in the middle of that window. This alternative approach might be more robust for poor maps. The model density could further be downweighted away from the center so that the center is emphasized. Using a larger block might improve the stability of the search, allowing for instance rigid-body refinement (including rotation) to be used, and potentially also allowing reliable shifts larger than 2 Å.

### 2.5. Applying smoothed offsets and refinement to create a morphed model with increased correlation with a density map

The final step in a cycle of the morphing process is to apply the smoothed shifts to each residue in the model and to refine the model. A single shift is applied to all of the atoms in a residue. This preserves the geometry of the residue and the orientation of the side chain. Adjacent residues will normally have different shifts, so that the geometry connecting them will generally be distorted. However, as the shifts are smoothed these distortions will typically be small. Although the morphed model will usually have very poor geometry, it can be refined to improve its geometry and agreement with the crystallographic data (Afonine *et al.*, 2005 ▶). The range of mean coordinate shifts applied in this process for the structures examined here is 0.5–1.3 Å.

### 2.6. Electron-density maps for morphing

The choice of target electron-density map for morphing is quite important, as this map must have sufficient information to identify shifts in position of local regions of the model, but it also must be relatively unbiased by the model itself so that the shifts are accurate. Maps that might be suitable for this purpose include those with coefficients based directly on the starting model but with reduced bias (2*mF*_o_ − *DF*_c_; Read, 1986 ▶), as well as density-modified maps (Wang, 1985 ▶; Cowtan & Main, 1996 ▶; Abrahams, 1997 ▶; Terwilliger, 1999 ▶; Blanc *et al.*, 2004 ▶; Cowtan, 2010 ▶). Additionally, maps specifically designed to further reduce model bias can be used. These include composite OMIT maps with or without refinement or simulated annealing (Hodel *et al.*, 1992 ▶) and prime-and-switch density-modified maps (Terwilliger, 2004 ▶).

### 2.7. Iteration of the morphing process

After a cycle of morphing, the morphed template will normally have changes both at the overall level, where residues will have new relationships to each other, and at a local level, where the atoms within a residue may have slightly different arrangements after refinement starting from their new positions. As the morphed template may be a closer match to the map, additional cycles of morphing have the potential to further improve the template. Normally, the coordinate shifts during morphing decrease rapidly after the first cycle and only a few cycles of morphing (typically six) are necessary. Morphing has been tested at resolutions ranging from 1.7 to 3.2 Å (see Table 1 ▶), but the method could in principle be used at a variety of resolutions, with corresponding adjustments in the radius of the sphere of density considered in the process (*r*_morph_) and also possibly in the size of the units to be tested for coordinate shifts (one residue in the current approach; potentially a group of residues at lower resolution).

### 2.8. Relationship between morphing and existing methods

Morphing is related to many existing methods from real-space rigid-body refinement (see, for example, Booth, 1947 ▶; Yeates & Rees, 1988 ▶; Afonine *et al.*, 2009 ▶) to deformable elastic network (DEN) refinement (Schröder *et al.*, 2007 ▶, 2010 ▶) and jelly-body refinement (Murshudov *et al.*, 2011 ▶), to procedures for finding fragments of structure in electron-density maps (Kleywegt & Jones, 1997 ▶; Cowtan, 1998 ▶; Terwilliger, 2001 ▶) and to normal-mode refinement (see, for example, Kidera & Go, 1992 ▶; Suhre & Sanejouand, 2004 ▶; Poon *et al.*, 2007 ▶; Delarue, 2008 ▶). Morphing shares the feature of moving a group of atoms all together with rigid-body refinement and finding fragments of structure in density maps. It differs from both in that the shift in coordinates estimated from a group of atoms centered at one atom (C^α^ or C_1_′ carbon, for example) is applied to just the atoms in that one residue rather than to all of the atoms used to identify the coordinate shift. It shares the capability of deforming a model with DEN, jelly-body refinement and normal-mode refinement. Unlike these methods it does not use a gradient, so the final shift could in principle escape from a local minimum. Morphing and normal-mode refinement differ from rigid-body jelly-body refinement in that shifts that are large can potentially be made (although normally only small shifts of up to 2 Å are considered in our process for morphing). Morphing is related to normal-mode refinement in that both are methods for identifying conformational differences between structures. They differ in that normal-mode refinement uses physical properties of the starting model to identify potential protein motions and tests the resulting coordinate shifts against crystallographic data, while in morphing coordinate shifts are identified from the electron-density map. Morphing also differs from all these methods in that it allows significant distortions to be made in the model (most of which are hopefully corrected during refinement). This potentially allows the approach to overcome geometrically unfavorable barriers between the starting conformation and the correct conformation.

### 2.9. Structure comparisons

Two methods are used here to quantify main-chain co­ordinate differences between structures: the r.m.s.d. and the percentile-based spread (Pozharski, 2010 ▶). The r.m.s.d. between two structures gives a measure of the overall differences between the structures and is the standard measure of these differences. In some cases, however, the largest coordinate differences, which tend to dominate the r.m.s.d., may not be as important as moderate ones. This is true of molecular replacement; an approximation to the likelihood score used in *Phaser* (Read & Chavali, 2007 ▶) shows that once errors are significantly greater than the resolution of the diffraction data (*d*_min_) structure-factor agreement will not be degraded further by making the errors even larger. Consequently, it is useful to consider in parallel other measures that are based on more typical parts of the structures. A percentile-based measure is useful in this role because it is insensitive to the values of either large or small differences. One possibility is to choose the median. In the present context, this would be the median value of the distances between corresponding atoms in two structures. This is essentially the value of the distances at the 50th percentile of the distribution. However, we choose to use the 60.8th percentile of the distances (Pozharski, 2010 ▶) here because it has the same expected value as the r.m.s.d. if the distances are derived from a three-dimensional Gaussian distribution.

In calculations of coordinate changes between pairs of structures, the choice of what pairs of atoms to compare can have a large effect. As the identity of each atom is likely to be less important than the coordinates of that atom in determining the utility of a structure in the early stages of structure determination, in this work differences are calculated between each main-chain atom in the structure being evaluated and the nearest main-chain atom in the comparison structure.

## 3. Results and discussion

### 3.1. Example of applying morphing to a structure

**Figure 1:**
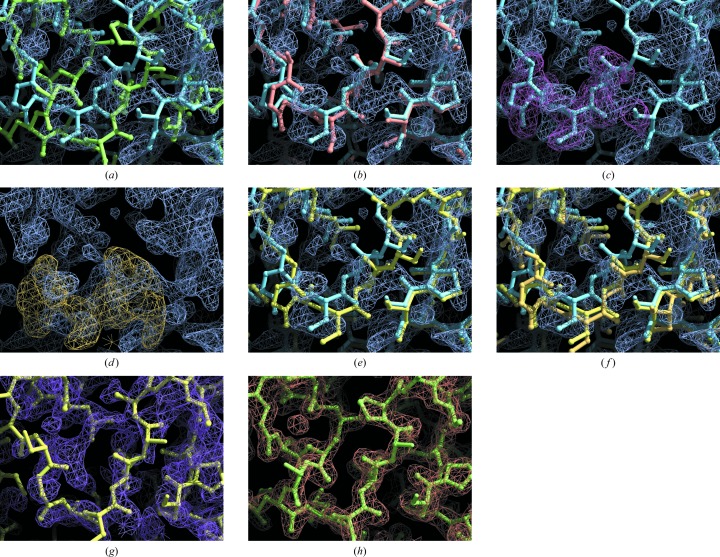
Application of morphing to the cab55348 structure. (*a*) Cip2 template (Pokkuluri *et al.*, 2011 ▶) in blue; final model of cab55348 in green; prime-and-switch electron-density map based on the template structure in purple. (*b*) Cip2 template and map as in (*a*); template after refinement with *phenix.refine* in orange. (*c*) Cip2 template and map as in (*a*); model density calculated from Cip2 template in purple. (*d*) Cip2 map as in (*a*); model density calculated from Cip2 template, offset to optimally match map, in purple. (*e*) Cip2 template and map as in (*a*); morphed Cip2 model in yellow. (*f*) Cip2 template, map and morphed Cip2 model as in (*e*); refined morphed Cip2 model in off-yellow. (*g*) Refined model after six cycles of morphing in yellow; prime-and-switch map based on model from cycle 5 of morphing in purple. (*h*) Automatically rebuilt model in green and density-modified electron density map in blue obtained starting from the map and model in (*g*). Contour levels in all the maps are at 1.5σ except for the model densities in (*c*) and (*d*), which are at 3.5σ. Figures were created with *Coot* (Emsley *et al.*, 2010 ▶) and *Raster*3*D* (Merritt & Bacon, 1997 ▶). A full *Coot* scene with all models and maps is available as supplementary material.

Fig. 1 ▶ illustrates the process of morphing a structure based on fit to an electron-density map. The target structure is one of those determined in a recent study using a combination of *Rosetta* structure modeling and crystallographic model building (cab55348; target 5 of DiMaio *et al.*, 2011 ▶; Table 1 ▶). The starting model was the structure of the glucuronoyl esterase Cip2 (PDB entry 3pic; Pokkuluri *et al.*, 2011 ▶), which was placed in the crystallographic unit cell of the target structure with *Phaser* (McCoy *et al.*, 2007 ▶) with non-matching segments deleted and non-identical side chains trimmed beyond their C^β^ atoms, yielding a template containing 354 residues and having a sequence identity to the target of 32%. When the residues in the aligned template are superimposed on the final target structure (DiMaio *et al.*, 2011 ▶) the main-chain atoms in the template and target differ by an r.m.s.d. of 2.10 Å and a percentile-based spread of 1.75 Å (Table 1 ▶). As described in §[Sec sec2]2, we use the 60.8th percentile of distances (the percentile-based spread; Pozharski, 2010 ▶) as a measure of the similarity of structures emphasizing the contribution of typical differences. This complements the use of the r.m.s.d., which emphasizes the contributions of large differences. The resolution of the crystallographic data was 1.9 Å.

In the previous work beginning with this template, standard model-building algorithms [*phenix.autobuild* (Terwilliger *et al.*, 2008 ▶) and *ARP*/*wARP* (Langer *et al.*, 2008 ▶; Cohen *et al.*, 2008 ▶)] were unsuccessful at rebuilding this model, yielding free *R* values of over 0.50 (DiMaio *et al.*, 2011 ▶). However, the structure could be built at that time with *phenix.autobuild* in combination with each of several recent methods including *Rosetta* modeling with density, an extreme version of multi-start simulating annealing (Hodel *et al.*, 1992 ▶) using 1000 attempts and DEN refinement without a grid search (Schröder *et al.*, 2010 ▶), leading to free *R* values of 0.31, 0.24 and 0.39, respectively (DiMaio *et al.*, 2011 ▶). Furthermore, our recent work on the refinement of the hp3342 structure suggests that the DEN refinement results could have been improved even further by carrying out a full grid search in DEN refinement prior to subsequent model building by *phenix.autobuild* (Brunger *et al.*, 2012 ▶). Additionally, a more recent version of *phenix.autobuild* can partially build this structure, yielding a free *R* value of 0.41 (*cf.* Table 3).

Fig. 1 ▶(*a*) shows a region of the Cip2 template structure along with the structure of the final refined model. In this region these structures are offset by about 1–3 Å. A prime-and-switch electron-density map based on the Cip2 template is also shown. This electron-density map agrees poorly with the Cip2 template (the correlation to a map calculated from the Cip2 template is 0.28) but it can still be used (see below) to identify appropriate distortions of the template that can make it more similar to the target structure.

As a reference for comparison with the results of morphing, the Cip2 template structure was refined with *phenix.refine* using individual coordinate refinement, individual isotropic thermal displacement parameters, automatic water placement and defaults for other parameters, including the number of cycles (three cycles). Fig. 1 ▶(*b*) shows the initial and refined Cip2-based models along with the prime-and-switch electron-density map. The refined template main-chain coordinates were slightly closer to the final model than the template, with the r.m.s.d. reduced from 2.10 to 2.03 Å. The percentile-based spread of the refined model decreased more substantially than the r.m.s.d. (from 1.75 to 1.54 Å), indicating that, as expected, the refinement process has improved the coordinates of those atoms that are closer to the target structure more than those that are far away.

Figs. 1 ▶(*c*)–1 ▶(*e*) illustrate the morphing process, demonstrating how the coordinate shift for residue 181 of the Cip2 template is obtained. Fig. 1 ▶(*c*) shows model density calculated from the initial Cip2 template superimposed on the prime-and-switch map. Fig. 1 ▶(*d*) shows the offset of this model density (a shift of 1.4 Å towards the lower right corner of the figure) that optimizes the correlation between these two maps. Fig. 1 ▶(*e*) shows the morphed template obtained by smoothing the coordinate shifts for the entire structure using a window of 11 residues and applying the smoothed shifts to each residue in the template, and Fig. 1 ▶(*f*) shows this morphed template after refinement. The correlation between the morphed model and the electron-density map was then 0.38, which is higher than for the initial model (0.28). The r.m.s. coordinate difference between the main-chain atoms of the refined morphed template and the final structure was 1.93 Å (percentile-based spread of 1.11 Å), which is considerably closer than for simple refinement.

Calculating a new prime-and-switch map each cycle and repeating the procedure in Figs. 1 ▶(*c*)–1 ▶(*e*) six times led to a refined morphed template (Fig. 1 ▶ *g*) that differed from the final refined model of the target structure (DiMaio *et al.*, 2011 ▶) by a main-chain atom r.m.s.d. of 1.86 Å and a percentile-based spread of 0.60 Å. The morphed model (Fig. 1 ▶ *g*) could be rebuilt automatically using *phenix.autobuild*, leading to a model (Fig. 1 ▶ *h*) that is essentially identical to the final refined model (main-chain r.m.s.d. of 0.34 Å for 393 residues) with an *R* value of 0.18 and a free *R* value of 0.22 (*cf.* Supplementary Table 1^1^).

### 3.2. Application of morphing to challenging molecular-replacement templates

We applied the morphing procedure to a set of 13 structure-determination problems that had been examined in detail in recent work combining structure modeling with crystallo­graphic model building as described above (Table 1 ▶; DiMaio *et al.*, 2011 ▶). In each case the starting point was a model that had been edited and placed in essentially the correct location in the crystal. The utility of morphing was further examined by using the morphed structures as a starting point for automated model building with *phenix.autobuild* and comparing the *R* values and free *R* values obtained with those obtained starting with the placed templates. To begin this analysis, we examined the utility of various methods for creating electron-density maps for use in morphing.

### 3.3. Comparison of various types of maps for use in morphing

Morphing is dependent on the availability of a relatively unbiased map that is of sufficient quality for the extraction of useful positional information about groups of atoms. It was not obvious *a priori* what type of map would be best for this purpose, so we carried out a systematic analysis of the effectiveness of morphing using several different types of maps that could be suitable. In each case the quality of a morphed model was evaluated by calculating the correlation of the resulting 2*mF*_o_ − *DF*_c_ (Read, 1986 ▶) electron-density map with the best available map for that structure (*cf.* Table 1 ▶; in most cases these were essentially final refined maps, but in several cases the structures have not yet been completed).

Table 2 ▶ lists the final 2*mF*_o_ − *DF*_c_ map correlations obtained for each of the 13 structures examined in previous work (DiMaio *et al.*, 2011 ▶) using four different types of maps in the morphing process. These maps were (i) 2*mF*_o_ − *DF*_c_ maps (Read, 1986 ▶), (ii) density-modified maps calculated with statistical density modification (Terwilliger, 1999 ▶), (iii) composite OMIT 2*mF*_o_ − *DF*_c_ maps (Hodel *et al.*, 1992 ▶) and (iv) prime-and-switch density-modified maps (Terwilliger, 2004 ▶). Additionally, a fifth procedure was carried out in which the morphed model produced using prime-and-switch maps was used as the starting point for a second round of morphing. For each structure, morphed models were compared with models obtained by refinement with *phenix.refine* using three macrocycles of atomic refinement.

Table 2 ▶ shows that any of the five procedures for morphing yielded very substantial improvements in nearly all of the 13 test structures. On average, the 2*mF*_o_ − *DF*_c_ maps obtained after refinement (without morphing) had a correlation with the best available maps of 0.493. Morphing with any of the map types yielded much higher average correlations of at least 0.672. Using 2*mF*_o_ − *DF*_c_ maps in morphing was effective, with an average map correlation after morphing of 0.672. Using density-modified maps and composite OMIT maps resulted in improved models (average map correlation of 0.685–0.690), and prime-and-switch maps resulted in further improvements (average map correlation of 0.699). As the prime-and-switch maps yielded the most model improvement, we used this map type in a test of whether further cycles would improve the morphing process. Iterating the entire process (doubling the number of cycles) did improve the models, yielding an average map correlation of 0.718 for the 13 test structures (Table 2 ▶).

Table 2 ▶ further shows that there was some variability in the amount of model improvement obtained using this morphing procedure. Models of lower starting quality were typically improved more than those of high starting quality. The most dramatic improvement was for the radA intein structure (Lyskowski *et al.*, 2011 ▶). The template used in this case was an automatically generated (and preliminary) NMR model of the same protein as in the crystal structure (DiMaio *et al.*, 2011 ▶). The 2*mF*_o_ − *DF*_c_ map calculated from refinement of the starting template had a correlation to the final map for this structure of only 0.299, while the model obtained after morphing yielded a map with a correlation of 0.826.

We also examined the free *R* values of models obtained by morphing. Supplementary Table 1^1^ lists free *R* values for models obtained in each of the tests listed in Table 2 ▶. Overall, the free *R* values were consistent with the map correlations. The mean free *R* value for the 13 structures after initial refinement of the templates was 0.53. After morphing using 2*mF*_o_ − *DF*_c_, density-modified or OMIT maps, the mean free *R* value was 0.48. After morphing with prime-and-switch maps the mean free *R* was 0.47 and with iteration of morphing using prime-and-switch maps the mean free *R* value was 0.46.

### 3.4. Using models obtained from morphing as a starting point for automated model building

The models in Table 2 ▶ and Supplementary Table 1^1^ obtained from morphing based on prime-and-switch maps were used as a starting point for automated model building with *phenix.autobuild* (Terwilliger *et al.*, 2008 ▶). Table 3 ▶ compares the free *R* values obtained with *phenix.autobuild* beginning with morphed models with those obtained starting from refinement alone. Table 3 ▶ shows that in three of the 13 cases morphing dramatically improved the model-building process. For the thiod structure, for example, autobuilding beginning with the initial template resulted in a free *R* value of 0.54, while with prime-and-switch morphing a greatly improved structure with a free *R* value of 0.34 was obtained. Similarly, for the estan structure autobuilding yielded a free *R* value of 0.54, while morphing followed by autobuilding yielded a model with a free *R* value of 0.25. Finally, for the cab55348 structure autobuilding alone yielded a free *R* value of 0.41, while morphing followed by autobuilding yielded a structure with a free *R* value of 0.22.

In the remaining six cases with a resolution of about 2.7 Å or better, autobuilding both with and without morphing resulted in a model with a free *R* value of 0.39 or better. In the final four cases at resolutions lower than 2.7 Å autobuilding with and without morphing yielded models with free *R* values ranging from 0.42 to 0.54, with the morphing process having relatively little effect. Although model morphing did not appear to improve model building for these four lower resolution cases, Supplementary Table 1^1^ shows that the morphing process does improve the initial electron-density maps for each of these cases. For example, in the case of the hp3342 structure at a resolution of 3.2 Å morphing improved the correlation between the starting 2*mF*_o_ − *DF*_c_ map and one based on a nearly final structure of this protein (Brunger *et al.*, 2012 ▶) from 0.479 to 0.658. Consequently, it seems likely that the lack of improvement in the autobuilding process is more from the lower effectiveness of the autobuilding process at this resolution than from a lack of improvement of the model with morphing.

### 3.5. Comparing morphing with extensive refinement with *phenix.refine*

During our testing of morphing, we considered the possibility that the improvement found with morphing was simply a consequence of the larger number of cycles of refinement applied in morphing (typically 18 cycles) compared with standard refinement (three cycles). To investigate this, we compared morphing with extensive refinement applying 100 cycles in *phenix.refine*. Table 4 ▶ shows that extensive refinement can indeed improve most of these models quite substantially, but in general not as much as is obtained by morphing. On average, the refinement increased the map correlation of 2*mF*_o_ − *DF*_c_ maps to the best available maps from 0.493 to 0.661, while morphing increased it to 0.718. In one case (thiod) extensive refinement yielded essentially no improvement (the map correlation increased from 0.344 to 0.389), while morphing yielded a greatly improved structure (map correlation of 0.646).

**Figure 2:**
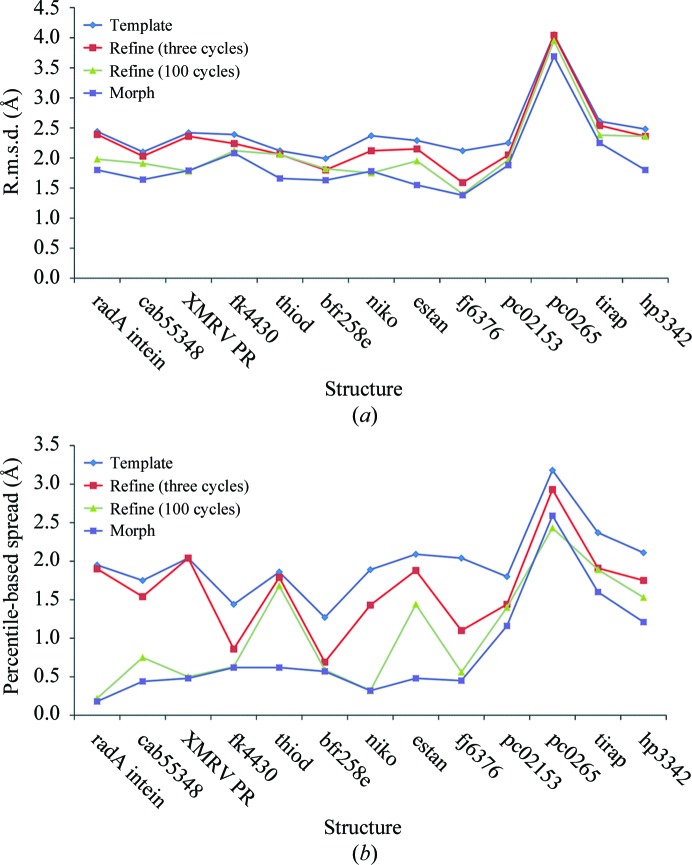
Differences between templates, refined and morphed models and the best available models for each structure. For each structure (listed along the *x* axis), the r.m.s.d. or percentile-based spread between the best available model for that structure and (i) the template, (ii) the template after three cycles of refinement with *phenix.refine*, (iii) the template after 100 cycles of refinement and (iv) the template after morphing using the prime-and-switch maps and iterating the morphing process for a total of 12 cycles is shown. (*a*) Differences calculated as r.m.s.d. (*b*) Differences calculated as percentile-based spread.

We compared extensive refinement and morphing further by examining the coordinate differences between the best available structures and each template, refined template, extensively refined template and morphed template. Fig. 2 ▶(*a*) shows the r.m.s.d. for each template before and after refinement and morphing and Fig. 2 ▶(*b*) shows the corresponding percentile-based spread values. Fig. 2 ▶ shows that for each of the structures standard refinement improved the models slightly, while extensive refinement improved them considerably more. In all cases except for pc0265 morphing gave lower or equal r.m.s.d. and percentile-based spread values compared with extensive refinement. In some cases (radA intein, XMRV PR, fk4430, bfr258e, niko, fj6376 and pc0265) the two procedures yielded very similar r.m.s.d. and percentile-based spread values. In others (cab55348, thiod, estan, pc02153, tirap and hp3342) morphing gave smaller values of each measure than extensive refinement.

Comparing Fig. 2 ▶(*a*) with Fig. 2 ▶(*b*), it can be seen that the relative improvements in percentile-based spread values are considerably more substantial than the improvements in r.m.s.d. For example, the percentile-based spread value for the starting template of the estan structure was 2.09 Å, which was reduced to 1.44 Å by extensive refinement and further to 0.54 Å by morphing (a 74% reduction in the percentile-based spread value). In contrast, the r.m.s.d. for this starting template was 2.29 Å, which was reduced to 1.95 Å by extensive refinement and to 1.55 Å by morphing (a 32% reduction in r.m.s.d.). The larger effects on percentile-based spread value compared with r.m.s.d. are consistent with the expectation that residues in the template that are very far from their positions in the final structure move only a little closer during morphing or refinement, while those that are only moderately far move much closer to the final structure.

### 3.6. Comparing morphing with *phenix.mr_rosetta*

The structures described in Table 1 ▶ and used here as tests of morphing have been extensively examined previously in tests of procedures for the combination of structure modeling with *Rosetta* and crystallographic model building (DiMaio *et al.*, 2011 ▶). We therefore compared the qualities of the models obtained with morphing and autobuilding with those obtained with *Rosetta* modeling and autobuilding using *phenix.mr_rosetta*. Additionally, we compared these with models obtained with extensive refinement followed by autobuilding. In the time since the previous work was carried out many improvements have been made in refinement with *phenix.refine* (*e.g.* the use of both real-space and reciprocal-space refinement and improved optimization of parameters). Consequently, we applied extensive refinement, morphing and *Rosetta* modeling followed by autobuilding, all with the current versions of *Phenix* (Adams *et al.*, 2010 ▶) and *Rosetta*. Supplementary Table 2^1^ shows the free *R* values obtained for each of the 13 structures using each approach. Most of the structures yield a free *R* value of 0.42 or better (the cutoff used in DiMaio *et al.*, 2011 ▶) with any of the three methods using current versions of these algorithms. However, only morphing and *Rosetta* modeling with autobuilding were sufficient to obtain a useful model for thiod (free *R* value of 0.55 for extensive refinement and autobuilding, free *R* values of 0.34 and 0.29 for morphing and for *Rosetta* modeling with autobuilding, respectively). Furthermore, *Rosetta* modeling with autobuilding yielded a considerably better model (free *R* value of 0.39) for pc02153 than either of the other two methods (free *R* values of 0.49 and 0.50). The computation required to carry out these analyses by extensive refinement is similar to that required for analyses using morphing, while using *Rosetta* modeling with autobuilding requires about ten times more computation (Table 4 ▶ and Supplementary Table 2^1^). We have also recently shown (Brunger *et al.*, 2012 ▶) that for rebuilding the hp3342 structure a combination of autobuilding, morphing and DEN refinement is superior to either autobuilding and morphing or DEN refinement alone.

### 3.7. Geometry and distorted models

The initial stages in the morphing process consist of deformation of a model without consideration of allowable geometries. Entire residues are moved as fixed units, so the junctions between residues are expected to have poor resulting geometry. This means that the initial morphed models are not entirely suitable for analysis or further model building without further modification. In our procedure, we include atomic refinement as part of each cycle of morphing, using the refinement process to restore realistic geometry where possible. When the overall morphing procedure is followed by iterative model building, density modification and refinement, as in procedures such as *phenix.autobuild*, the model-building process is expected to restore reasonable geometry to the model.

## 4. Conclusions

We find that morphing is quite powerful for improving the quality of models that principally differ from a target structure by simple deformations. The method may therefore be useful in a variety of situations encountered in macromolecular structure determination. In general, the procedure may be useful in any case where a model is available that differs at least in part through simple distortion of the target structure and an electron-density map can be obtained that contains information about how to change that model.

A clear application is that described in this work in which a search model has been placed in the crystallographic cell by molecular replacement but the model is not close enough to the target structure for automated model building to be effective. An extension of this would be to apply morphing to a series of potential molecular-replacement solutions and to identify the best based on the quality of the map or the free *R* value.

Another application would be the morphing of homology models into experimentally obtained electron-density maps. For example, a SAD-phased density-modified electron-density map might be of insufficient quality to build a satisfactory model but still be of high enough quality to be useful in morphing a distant homology model to match the density. This might be effective even in cases where the homology model was too distant to be successful in conventional molecular replacement or in MRSAD phasing including the homology model along with SAD phasing information (Schuermann & Tanner, 2003 ▶). The morphed model then could be used in MRSAD phasing or as a source of partial model information in iterative model-building procedures. Methods for morphing may also be useful in combination with other methods that take advantage of local similarities of homologous proteins, such as DEN and jelly-body refinement.

## Supplementary Material

Supplementary material file. DOI: 10.1107/S0907444912015636/kw5044sup1.pdf

Coot scene.. DOI: 10.1107/S0907444912015636/kw5044sup2.tar.gz

## Figures and Tables

**Table 1:** Structures used in analysis of morphing

Structure	Resolution (Å)	Identity (%)	NCS copies	Chain length	Free *R* value†	Template chain length	Template r.m.s.d.‡ (Å)	Template percentile-based spread§ (Å)	Notes	ID in DiMaio *et al.* (2011 ▶)¶
radA intein	1.7	100	2	174	0.26	174	2.44	1.95	DiMaio *et al.* (2011 ▶); Lyskowski *et al.* (2011 ▶)	12
cab55348	1.9	31	1	420	0.24	354	2.10	1.75	DiMaio *et al.* (2011 ▶)	5
XMRV PR	2.0	30	2	133	0.23	97	2.42	2.04	PDB entry 3nr6; Li *et al.* (2011 ▶)	6
fk4430	2.1	22	1	205	0.29	132	2.39	1.44	DiMaio *et al.* (2011 ▶)	1
thiod	2.1	22/15††	1	248	0.26	214	2.12	1.86	DiMaio *et al.* (2011 ▶)	7
bfr258e	2.2	19	2	168	0.22	134	1.99	1.27	PDB entry 3nng; Northeast Structural Genomics Consortium (unpublished work)	2
niko	2.5	27	2	473	0.31	415	2.37	1.89	DiMaio *et al.* (2011 ▶)	3
estan	2.5	18	1	372	0.25	257	2.29	2.09	DiMaio *et al.* (2011 ▶)	11
fj6376	2.7	21	4	248	0.24	224	2.12	2.04	PDB entry 3o8s; Joint Center for Structural Genomics (unpublished work)	4
pc02153	2.8	29	1	312	0.38	287	2.25	1.80	DiMaio *et al.* (2011 ▶)	8
pc0265	2.9	29	2	343	0.23	308	4.05‡‡	3.18	PDB entry 3on5; Joint Center for Structural Genomics (unpublished work)	13
tirap	3.0	22	1	176	0.29	141	2.61	2.37	DiMaio *et al.* (2011 ▶)	9
hp3342	3.2§§	20	1	369	0.26	352	2.48	2.11	PDB entry 3tx8; Brunger *et al.* (2012 ▶)	10

† The free *R* value corresponds to deposited refined structures where available (as listed in the Notes column) and based on the available structures with lowest free *R* value in other cases (taken from DiMaio *et al.*, 2011 ▶). The best available maps used here were 2*mF*_o_ − *DF*_c_ maps based on these structures.

‡ Template r.m.s.d. is calculated between main-chain atoms of the template and the nearest main-chain atoms of the final structure used for the free *R* value and map calculations.

§ Percentile-based spread (Pozharski, 2010 ▶) is the distance corresponding to the 60.8th percentile of distances between main-chain atoms of the template and final structure.

¶ These structures and the template structures used as starting models are the same as those used in DiMaio *et al.* (2011 ▶) and are referred to in that work with an ID number instead of a name. The starting model for the radA intein structure was a preliminary NMR model created with an automatic procedure. The starting model for thiod consisted of a *Rosetta* model for one domain and a molecular-replacement solution for the other. The starting models for the other structures were edited homology models placed in the unit cell either by molecular replacement or by superposition on a molecular-replacement solution as described in DiMaio *et al.* (2011 ▶).

†† Separate templates were used to model the two domains of thiod; the template for one domain had a sequence identity of 22% and the other had an identity of 15%.

‡‡ The pc0265 structure has two domains and the relative positions of these domains differ in the template and the target structure.

§§ The resolution of the data used here for the hp3342 structure was 3.2 Å. This is the same data (non-anomalous data from inflection point) that was used previously (DiMaio *et al.*, 2011 ▶) and that was used in early stages of the recent full determination of this structure (Brunger *et al.*, 2012 ▶).

**Table 2:** Map correlation to best available maps for various morphing strategies

		Morphing with various maps				
Structure	Refinement	2*mF*_o_ − *DF*_o_	Density modified	OMIT	Prime-and-switch	Prime-and-switch (repeated)†
radA intein	0.299	0.826	0.866	0.861	0.853	0.876
cab55348	0.361	0.595	0.600	0.619	0.649	0.684
XMRV PR	0.304	0.747	0.740	0.746	0.733	0.712
fk4430	0.690	0.703	0.704	0.706	0.690	0.715
thiod	0.344	0.477	0.498	0.586	0.548	0.646
bfr258e	0.667	0.685	0.700	0.691	0.702	0.708
niko	0.535	0.788	0.785	0.790	0.785	0.790
estan	0.376	0.571	0.634	0.612	0.671	0.680
fj6376	0.637	0.764	0.769	0.763	0.761	0.769
pc02153	0.708	0.771	0.774	0.760	0.785	0.783
pc0265	0.443	0.576	0.553	0.569	0.575	0.603
tirap	0.567	0.659	0.684	0.664	0.694	0.715
hp3342	0.479	0.571	0.597	0.596	0.645	0.658
Mean	0.493	0.672	0.685	0.690	0.699	0.718

† The models from the prime-and-switch map-based morphing were used as the starting point for a second round of morphing using prime-and-switch maps. A single prime-and-switch map was calculated at the beginning of this second round of morphing and was used for the entire round.

**Table 3:** Free *R* values after morphing and autobuilding

Structure	Autobuild free *R*	Morphing and autobuild free *R* †
radA intein	0.29	0.29
**cab55348**	0.41	**0.22**
XMRV PR	0.39	0.37
fk4430	0.34	0.33
**thiod**	0.54	**0.34**
bfr258e	0.28	0.27
niko	0.29	0.29
**estan**	0.54	**0.25**
fj6376	0.29	0.31
pc02153	0.48	0.49
pc0265	0.43	0.42
tirap	0.45	0.51
hp3342	0.54	0.51

† Morphing using prime-and-switch maps and a total of six cycles, as in Table 2 ▶. Cases in which morphing reduced the free *R* value by 0.2 units or more are shown in bold.

**Table 4:** Map correlation to the best available maps for extensive *phenix.refine* refinement compared with morphing

Structure	Refinement (three cycles)	Refinement (100 cycles)	Morphing†
radA intein	0.299	0.840	0.876
cab55348	0.361	0.607	**0.684**
XMRV PR	0.304	0.717	0.712
fk4430	0.690	0.693	0.715
thiod	0.344	0.389	**0.646**
bfr258e	0.667	0.678	0.708
niko	0.535	0.788	0.790
estan	0.376	0.569	**0.680**
fj6376	0.637	0.757	0.769
pc02153	0.708	0.743	0.783
pc0265	0.443	0.611	0.603
tirap	0.567	0.648	**0.715**
hp3342	0.479	0.552	**0.658**
Mean	0.493	0.661	0.718

† Morphing using prime-and-switch maps and a total of six cycles, as in Table 2 ▶. The cases where morphing improved the correlation compared with 100 cycles of refinement by 0.05 units or more are shown in bold. The CPU time (using 2.9 GHz Intel Xeon processors) required to run 100 cycles of refinement varied from 1 to 5.5 h for these structures (mean of 2.6 h) and the CPU time required to run morphing ranged from 0.5 to 5 h (mean of 2.0 h).

## Footnotes

1 Supplementary material has been deposited in the IUCr electronic archive (Reference: KW5044). Services for accessing this material are described at the back of the journal.
