# Supplementary material for: Improved crystallographic models through iterated local density-guided model deformation and reciprocal-space refinement
Source: Acta Crystallogr D Biol Crystallogr. 2012 Jun 19;68(Pt 7):861–70. doi: 10.1107/S0907444912015636 (PMC3388814; doi:10.1107/S0907444912015636)
Supplement: Supplementary file 1 [file d-68-00861-sup1.pdf]

Supplementary Material

Supplementary Table 1. Free R values for morphing strategies

		Morphing with various maps				
**Structure**	**Refinement**	**2mFo-DFc**	**density-modified**	**Omit**	**Prime-and-switch**	**Prime-and-switch (repeated)**
radA intein	0.552	0.395	0.363	0.376	0.380	0.359
cab55348	0.531	0.497	0.506	0.489	0.484	0.462
XMRV PR	0.583	0.521	0.534	0.518	0.537	0.545
fk4430	0.470	0.443	0.457	0.456	0.430	0.442
thiod	0.575	0.561	0.568	0.560	0.545	0.525
bfr258e	0.507	0.488	0.488	0.489	0.485	0.480
niko	0.516	0.420	0.419	0.412	0.413	0.414
estan	0.549	0.518	0.510	0.525	0.485	0.489
fj6376	0.470	0.436	0.434	0.437	0.434	0.429
pc02153	0.528	0.483	0.502	0.506	0.488	0.498
pc0265	0.539	0.503	0.495	0.515	0.499	0.491
tirap	0.511	0.487	0.469	0.486	0.509	0.436
hp3342	0.540	0.501	0.504	0.512	0.478	0.470
**MEAN**	**0.529**	**0.481**	**0.481**	**0.483**	**0.474**	**0.465**

Supplementary Table 2. Free R values after extensive refinement and autobuilding compared to morphing and autobuilding and to *phenix.mr_rosetta*

**Structure**	**Extensive refinement and *Autobuild* free R**	**Morphing and *Autobuild* free R***	***mr_rosetta* free R***
radA intein	0.29	0.29	0.28
**cab55348**	0.23	0.22	0.25
XMRV PR	0.35	0.37	0.31
fk4430	0.32	0.33	0.31
**thiod**	0.55	**0.34**	**0.29**
bfr258e	0.26	0.27	0.27
niko	0.29	0.29	0.28
**estan**	0.25	0.25	0.25
fj6376	0.29	0.31	0.29
pc02153	0.50	0.49	**0.39**
pc0265	0.39	0.42	0.39
tirap	0.47	0.51	0.45**
hp3342	0.52	0.51	0.48

*Cases where free R-values for morphing and autobuilding or *mr_rosetta* are lower than extensive refinement alone by 0.1 unit or more are in bold. The CPU time required for the *mr_rosetta* analyses typically ranged from 30 to 130 hours (DiMaio et al., 2011).

**For the tirap structure the starting model for refinement, morphing and autobuilding was a model consisting only of Ser, Gly and Ala (DiMaio et al., 2011). As this template was not suitable for *mr_rosetta* analysis (which requires a sequence alignment) the starting model used for *mr_rosetta* was an edited homology model (Rabeh et al, 2006; PDB entry 2H16, Berman et al., 2000), after placement with Phaser (Read, 2001).

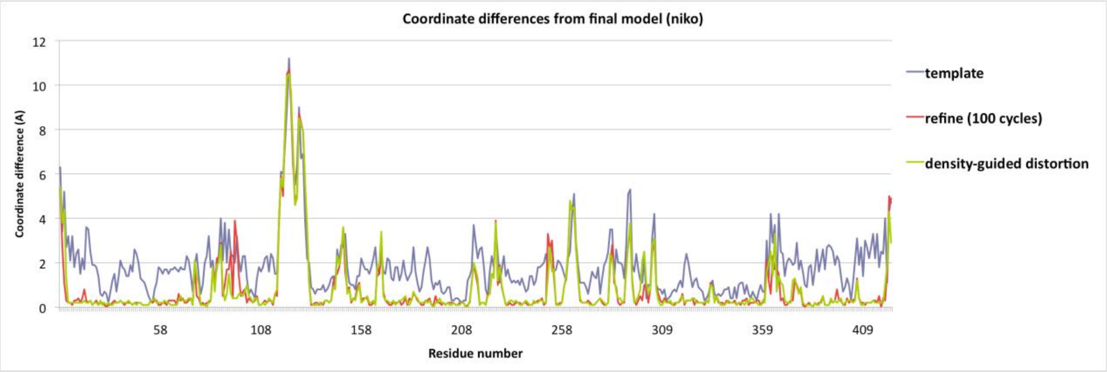

A

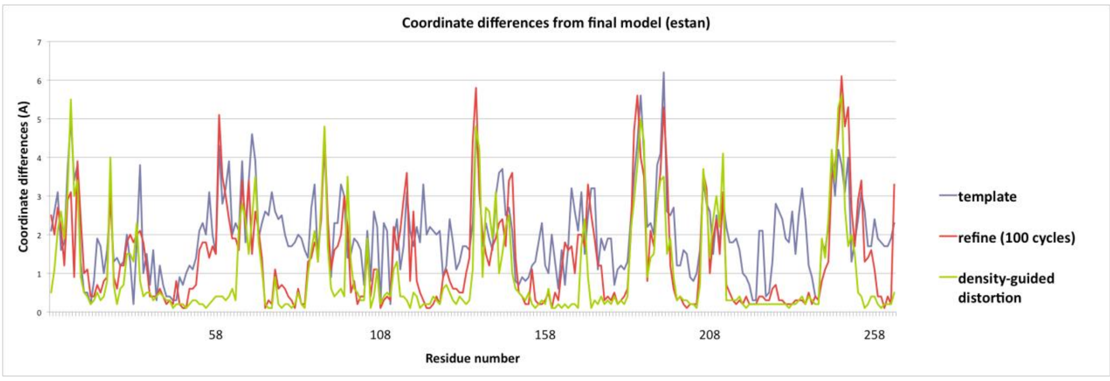

B

Supplementary Figure 1. Residue-based coordinate differences between models for the *niko* and *estan* structures and the final models for these structures are shown. In each case the distance between each C_α atom in a model and the closest C_α atom in the final corresponding refined model is plotted as a function of residue number. The models compared are the same models that are considered in Fig. 2 for these structures. The models analyzed are the initial templates (after placement in the correct position in the unit cell), the templates after 100 cycles of refinement, and the templates after morphing using prime-and-switch maps. A. Coordinate differences for the *niko* model. B. Coordinate differences for the *estan* model.
